# Classification of Juvenile Delinquents
*A paper read at the Child Psychiatry Section of the Royal Medico-Psychological Association, 11th May 1963.


**Published:** 1963-10

**Authors:** Frank Bodman


### CLASSIFICATION OF JUVENILE DELINQUENTS*

BY

FRANK BODMAN, M.D., D.P.M.

The Regional Board has allotted me three sessions a week at the Kingswood Classifying School. I find that to make a psychiatric assessment takes an hour for each case, including a preliminary short discussion with the Psychologist, and the reading of the files from Probation Officers, Child Guidance Clinics and Approved Schools. Some 9 boys a week can therefore be seen, or approximately 450 boys a year.

As the intake at this Classifying School is some 860 boys a year, not every boy is seen by the Psychiatrist; certain boys are referred to him for an opinion by the Psychologist. These are boys who are considered mentally disturbed or subnormal, or who present special problems, whether educational, social or medical.

When I arrive at the school, I find on my desk a pile of files relating to the boys selected for me to see. The Psychologist joins me, and indicates the special features in the information available about each boy, or in the tests that he had carried out, which indicated to him the need for my opinion.

The collected information will include the results of a battery of intelligence and projection tests, which may show some unusual anomaly or discrepancy. There will also be the report of the Social Workers' interview with the boy, and with the parents if they have visited. There will be reports from the class teachers and instructors, reports from the Probation Officer to the Juvenile Court, and his Record of Supervision, if the boy has been on probation. In addition, there may be a bulky file from the Child Guidance Clinic, and perhaps a file from a previous Approved School. This can entail a formidable amount of reading, but I consider it is essential, if one is to understand why the boy has failed to respond to probation, to therapy at the clinic, or to training at a previous Approved School.

I like to plot out the information in an annuary of the boy's life, and see if any pattern emerges. Was the boy a problem even at the age of 3, wandering away from home? Did his anti-social behaviour begin when the family moved to a new council estate or a new city? Was he well-behaved until he transferred to Secondary Modern School? Parents often like to attribute delinquency to an accident involving a head injury, but by plotting out the record, it may often be shown that misconduct was serious long before the accident.

I usually begin the interview by taking the boy through the Vineland Social Maturity Test. The questions involved cover most of the working-life and leisure occupations, and serve as a useful introduction. The test has been standardized and the final result is the boy's social age and social quotient. These figures can be compared with the boy's mental age and intelligence quotient. The majority of delinquent boys are socially immature and the average social quotient is under 81. Their characteristic failures in this test are in items related to self-direction and self-occupation.

When there is a large discrepancy between I.Q. and S.Q. of over 30 points, serious constitutional instability is probable. This may be a psychopathic instability or an instability associated with early schizophrenia. Occasionally, one finds a social quotient higher than the intelligence quotient. This usually indicates that the boy's limitations have not been recognized, and that more responsibility has been expected

* A paper read at the Child Psychiatry Section of the Royal Medico-Psychological Association, 11th May 1963.

of him than his capacity warrants. Such a boy may find relief of tension in aggressive and destructive behaviour.

The next stage is to find out something of this boy's outstanding character traits. Here I use as a framework Glueck's prediction tables. In the table drawn up for the psychiatric interview, the Gluecks have isolated the factors of:—Adventurousness, Extroversion, Suggestibility, Stubbornness, Emotional Disharmony. This combination of traits proved characteristic of delinquents in the Gluecks' controlled studies.

We all are familiar with boys whose mothers complain they are too daring, who act impulsively; on whom the Probation Officers sadly comment that they are too easily led; whose fathers grumble that their sons are too pigheaded and won't listen to reason; and of whom everyone moans that they are so inconsiderate. Not every boy shows all these qualities, but when they are exhibited in one individual they form a potentially antisocial combination. The Gluecks have worked out weighted scores for each of these character traits and established a deadline, above which it is likely that the testee will persist in delinquent behaviour

Next in the interview, comes the investigation of the boy's environment, of which the most important elements are the family attitudes. This has already been covered to a large extent by the Probation Officers reports and records, and by the Child Guidance Team, as well as by the interview of the Classifying School's Social Worker with the boy and his parents.

I find it useful however to ascertain the boy's feelings about his family which may be very different from the family's assessment. There again the Gluecks have elucidated from their researches a valuable guide. I therefore ask the boy what he thinks of his father's discipline—is it fair or unfair, easygoing or harsh, consistent or inconsistent? Does his mother make plans for his leisure time? Does she want to know where he is going when he goes out? Does she approve or disapprove of his boy or girl companions? Does he take any notice of her warnings? Does he think of his father as interested in his progress or achievements or does he think of his father as indifferent or critical? Does he think of his mother as indulgent and sympathetic? Is she fussy or over protective? Or does he speak of her as always moaning or nagging? What about the family as a whole? Is there a strong we-feeling? Do they take a pride in their home and each other? Do they share interests or each go their own way? Has the family begun to disperse or break up? Has an admired elder brother left home to be married or to join the Services? The parents, have they separated or divorced? Has home just become a convenience—a place to hang your hat?

The most damaging factors, according to the Gluecks, are firstly the harsh sergeant major type of paternal discipline; then the mother who is lax in her supervision and leaves her sons to their own devices; then the mother who openly rejects her son—and most damaging of all, the home which the boy only frequents as a lodging house—a place in which to eat and sleep.

We are often asked about the delinquent boy, "How much is his behaviour due to his environment, how much to his essential personality". While I do not attach great importance to the exact figures in the scores, some estimate can be made by comparing the personality score with the social background score.

Not only must an attempt be made to visualize the boy in his family setting, but some attempt must be made to envisage this family in the community in which they are living. Here the Probation Officer's reports are valuable. The family may be accepted in the community or rejected. They may have lived in their town or city all their lives, and their grandparents before them; or in contrast they may be one of these mobile families, where the breadwinner moves around, because he quarrels with his employer, or seeks ever better wages. North-countrymen come South and find the southerners soft; East Anglians move West and are homesick. Families climb up the social ladder, keep up with the Joneses, and find difficulty in acclimatising to different codes of manners and behaviour. Other families are large and are sliding down the social ladder.

There is the broken home, the mother who deserts and leaves the father to bring up the children, perhaps with the aid of the grandparents, the father fed up with his responsibilities, irresponsibly cohabits with another woman, who perhaps has children of her own, and who rejects these acquired foster children.

In between are the rough families, with no social aspirations and no member of sufficient intelligence to gain a grammar school place, the father a semi-skilled or unskilled worker; or the dull families, where one or more members are educationally subnormal (often these families are large).

By this time, it should be possible to see some pattern emerging of the boy in his family and community, and it is time to plan recommendations about training and treatment. Here I find Peter Scott's classification useful.

Some boys have been trained in anti-social behaviour. They come from homes where anti-authority attitudes are the norm; where the boy is brought up to think of the policeman as an enemy; where the door is shut in the face of the health visitor; where the father goes to prison rather than pay for the boy's maintenance; where the mother harbours her absconding son. Such a boy, brought up in such a home, may have a normal personality. He is doing what is usual in his family and district. He will feel no guilt about his behaviour, and his family and mates will not be critical; such a boy's treatment is retraining. He is not in need of psychotherapy.

Much more frequently I see the boy who is untrained. There have been no steady standards of behaviour provided, no inducements to learn, no examples to imitate. The parents have been self-indulgent, inconsistent, on the make. Parental management has been unpredictable because of friction and dissension. The boy has never learnt to control his impulses, is confused by arbitrary punishments, and has learnt to play off one parent against the other. This boy also is in need of training, patient supervision, and when occasion demands it, appropriate sanctions. Psychotherapy is very rarely indicated as the boy's character is too unformed, the personality too poorly integrated to be able to make use of it.

The delinquents who need some form of psychotherapy are those boys whose behaviour is compensatory. These boys are reacting to difficulties in their environment, or handicaps in their development. They often have a feeling of inferiority or inadequacy. They save their faces by adopting an arrogant "couldn't care less" manner. They cannot tolerate criticism; they think of themselves as "tough", and take pride in their offences. Having built up this pseudo persona, they cannot abandon it and delinquent patterns are established. They will resist change, be unwilling to abandon these defensive attitudes, and while they will require retraining, some psychotherapy, whether individual or group, will be required to help them to face their inferiorities and inadequacies.

There remains a small group described by Peter Scott as boys with rigid fixations. These are boys whose very early life experiences have been so overwhelmingly frustrating that they are unable to learn. They respond to new or unknown situations with fixed or inappropriate responses. These offenders from a toddler age show stereotyped, stupid, purposeless patterns of behaviour. Long periods in a controlled environment do not alter them; punishment has no effect. The offences produce no material gain and do not make sense. It seems impossible to understand the behaviour or to sympathize with the offender. In this class are found some cases of arson, some examples of violent aggression, some persistent absconders. They are inaccessible to psychotherapy.

I prefer to write my report at once, before I see the next boy. It is easy to confuse impressions, and in psychiatry impressions, tone of voice, bearing, movements of hands and feet, facial expressions, are important. Even the way the boy leaves the room can be significant. He may say "goodnight" or "thank you", even offer to shake hands; he may sing or whistle as he runs back to the common room; he may stamp down the corridor swearing, or he may come back and ask me a question.

Psychiatry, even more than the rest of medicine, is an art as well as a science. We make use of scientific methods and techniques, but in the last resort, feeling and intuition must also be allowed to play their part.

